# Neo-sternum reconstruction using costal cartilage approximation and small Permacol® patch repair in the treatment of Cantrell pentalogy: a case report

**DOI:** 10.1186/s13019-015-0241-x

**Published:** 2015-03-25

**Authors:** Chang Wan Kim, Hyun Min Cho, Bong Soo Son, Do Hyung Kim

**Affiliations:** 1 Department of Thoracic and Cardiovascular Surgery, Pusan National University Yangsan Hospital, Geumo-ro 20, Beomeo-ri, Mulgeum-eup, Yangsan-si, Gyeongsangnam-do 626-770 Republic of Korea; 2 Department of Thoracic and Cardiovascular Surgery, Trauma Center of Pusan National University Hospital, 179, Gudeok-ro, Seo-gu, Busan, South Korea

## Abstract

The ideal treatment for pentalogy of Cantrell is neo-sternum reconstruction by using autologous tissues. Although treatment timing varies depending on the degree of deformity and patient’s condition, the principle is performing the procedure at the earliest, to prevent blunt or piercing trauma to the heart. However, the challenge is performing the procedure on a neonate, because feasibility of the procedure is affected by the size of the defect, and limitations in utilizable autologous tissues. We used a small biocompatible patch (Permacol®) and lower costal cartilage to perform curative neo-sternum reconstruction, which is a simple and safe treatment method.

## Background

Patients with pentalogy of Cantrell exhibit the following five characteristics: a midline supraumbilical abdominal wall defect, defect of the lower part of the sternum, deficiency of the anterior diaphragm, defect in the diaphragmatic pericardium, and congenital heart malformation [1]. The ideal treatment for pentalogy of Cantrell is neo-sternum reconstruction by using autologous tissues. Although the treatment timing varies depending on the degree of deformity and patient’s condition, the principle is performing the procedure at the earliest, to prevent blunt or piercing trauma to the heart. However, the challenge is performing the procedure on a neonate, because feasibility of the procedure is affected by the size of the defect and limitations in the amounts of utilizable autologous tissues [2]. We used a small biocompatible patch (Permacol®) and lower costal cartilage to perform curative neo-sternum reconstruction.

## Case presentation

**Figure 1:**
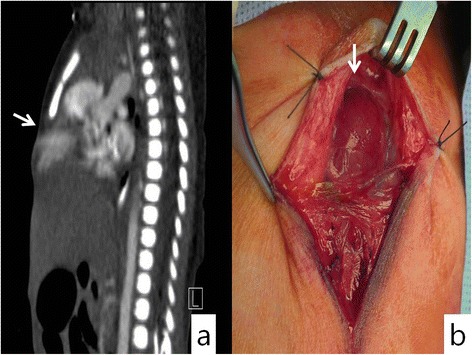
**Chest computed tomography (CT) of an infant with cardiac defect showing (a) protrusion of the cardiac apex through dehiscent chest wall and no ossification center at the lower sternum and (b) intra-operative findings showing four defects in the lower sternum, abdominal wall, anterior portion of the diaphragm, and diaphragmatic portion of the pericardium.**

A 3-cm palpable protruding mass, abdominal wall defect, and lower sternum defect were found in a female infant at 38 weeks and 1 day gestation. Chest and abdominal computed tomographies (CTs) as well as abdominal ultrasound showed lower sternal, pericardial, abdominal wall, diaphragm defects and protruding left ventricle (Figure 1a). Echocardiography led to the diagnosis of a small atrial septal defect (ASD) and patent ductus arteriosus (PDA). After delivery, the infant showed no other specific clinical symptom. Under the diagnosis of pentalogy of Cantrell, surgical procedure was performed at 10 days after the birth of the neonate.

**Figure 2:**
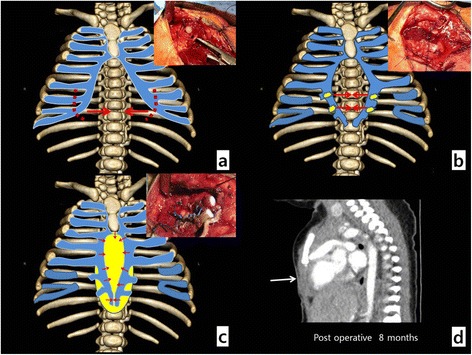
**Operative findings. (a)** Lowermost costal cartilage resection is performed for cartilage approximation; **(b)** Approximations of both costal cartilages are attempted, but the high tension of the string tying the cartilages prevents obtaining optimal results; **(c)** Permacol® patch of size similar to the defect is temporarily affixed onto the left costal cartilage by using a 3–0 Ethibond and cut into the appropriate size. The stitches on the right side is close to the left suture site to minimize the size of the Permacol® patch for the approximation of costal cartilages, and the bottom area of the patch is used to reinforce the diaphragm and peritoneum defect areas; **(d)** follow up CT shows no thoraco abdominal defect at the heart protruding site (white arrow).

The skin incision above the protruding heart was performed, and the hypoderm was separated from the heart. Because of the sternum defect, the rectus abdominis muscle was not attached to the center of the sternum. The left ventricle apex was protruded along the thoraco-abdominal wall defect, and parts of the pericardium and diaphragm were observed as defect (Figure 1b). First, chest wall reconstruction was attempted by separating the rectus abdominis muscle from the lower costal cartilage and resecting the lowermost costal cartilage to approximate the cartilages on both sides toward the medial side (Figure 2a). Because of the high tension of the string tied around the cartilages, the risk of amputating the cartilage was present. Moreover, simple approximation of the cartilage was insufficient for covering the protruding left ventricle (Figure 2b). Thus, a small patch repair was included for the chest wall reconstruction procedure. The Permacol® patch(1x4cm, Covidien) was temporarily affixed onto the left costal cartilage by using 3–0 Ethibond and cut into the appropriate size. The stitches on the right side were performed close to the left suture site to minimize the size of the Permacol® patch used. The Permacol® patch corner stitches allowed even distribution of the tension, thereby allowing close approximation of costal cartilage without tension (Figure 2c). The bottom area of the patch was used to reinforce the diaphragm and peritoneum defects. The pectoralis major muscle was attached to the rectus abdominis at the upper site of the chest wall reconstruction. After a drain was inserted, the wound was closed. The associated cardiac anomalies were not surgically repaired. The patient was discharged from the hospital without significant complications two weeks after the procedure. Currently, at eight months after the procedure, her condition is still being monitored carefully (Figure 2d).

## Comment

In general, two techniques are followed in congenital defect surgical repair: using autologous tissues or artificial materials. Repair by using autologous tissues is preferred to minimize the risk of infection and accommodate the growth during remodeling of the wall [3]. The ideal corrective procedure for a patient with lower sternal cleft, including pentalogy of Cantrell, is sternal reconstruction through approximation of low costal-cartilage by using autologous tissues and covering with the pectoralis major and rectus abdominis muscles [4]. To minimize the tension of the strings during the low costal approximation, sternum resection and lowermost costal cartilage resection should be performed, and tying for the approximation should be performed using encircling suture. However, full approximation of the cartilage is difficult with encircling sutures alone due to risk of amputating the cartilages because of the tension. Additionally, the technique of thoraco-abdominal repair by using autologous tissues is greatly affected by the defect size and corresponding autologous tissue size. Consequently, it is difficult to acquire sufficient amounts of autologous tissues [5]. These disadvantages are why sternal reconstruction by thoraco-abdominal wall repair, typically, is not performed on neonates despite its many advantages.

## Conclusions

We improved the existing surgical technique of sternal reconstruction in neonates to facilitate a safer and relatively quicker procedure, thereby overcoming several disadvantages of the technique. In addition to promoting the advantages of using autologous tissues, the new procedure helped minimize the tension of the string during the low costal cartilage approximation by using a small-sized biocompatible patch, which is affixed onto the resected left low costal cartilage by using interrupted stitch. Then, the contra-lateral stitch was performed close to the left stitch to facilitate the approximation of both cartilages. Thus, the tension resulting from approximation of the costal cartilages is distributed evenly on the patch, enabling close approximation with less tension. Further, the gap in the central area was covered using the patch. A minimal amount of the biocompatible agent Permacol® was used for the type and size of the patch, taking into account the risk of infection and mal-growth modeling of the thoraco-abdominal wall. Thus, patients who do not need corrective cardiac surgery could benefit from the safe and relatively quick option of thoraco-abdominal wall reconstruction by using a small patch and low costal cartilage resection and approximation.

## Consent

Written informed consent was obtained from the patient for the publication of this report and any accompanying images.
